# A user-friendly plug-and-play cyclic olefin copolymer-based microfluidic chip for room-temperature, fixed-target serial crystallography

**DOI:** 10.1107/S2059798323007027

**Published:** 2023-09-25

**Authors:** Zhongrui Liu, Kevin K. Gu, Megan L. Shelby, Deepshika Gilbile, Artem Y. Lyubimov, Silvia Russi, Aina E. Cohen, Sankar Raju Narayanasamy, Sabine Botha, Christopher Kupitz, Raymond G. Sierra, Fredric Poitevin, Antonio Gilardi, Stella Lisova, Matthew A. Coleman, Matthias Frank, Tonya L. Kuhl

**Affiliations:** a Department of Chemical Engineering, University of California at Davis, Davis, CA 95616, USA; b Biosciences and Biotechnology Division, Lawrence Livermore National Laboratory, Livermore, CA 94550, USA; c Stanford Synchrotron Radiation Lightsource, SLAC National Accelerator Laboratory, Menlo Park, CA 94025, USA; d Linac Coherent Light Source, SLAC National Accelerator Laboratory, Menlo Park, CA 94025, USA; e Department of Physics, Arizona State University, Tempe, AZ 85287, USA; f Department of Radiation Oncology, School of Medicine, University of California at Davis, Sacramento, CA 95817, USA; g Department of Biochemistry and Molecular Medicine, School of Medicine, University of California at Davis, Sacramento, CA 95817, USA; Stanford University, USA

**Keywords:** X-ray crystallography, synchrotrons, XFELs, sample delivery, fixed targets, cyclic olefin copolymer, microfluidics, serial crystallography, room-temperature SFX

## Abstract

Over the past two decades, serial X-ray crystallography has enabled the structure determination of a wide range of proteins. With the advent of X-ray free-electron lasers (XFELs), ever-smaller crystals have yielded high-resolution diffraction and structure determination. A crucial need to continue advancement is the efficient delivery of fragile and micrometre-sized crystals to the X-ray beam intersection. This paper presents an improved design of an all-polymer microfluidic ‘chip’ for room-temperature fixed-target serial crystallography that can be tailored to broadly meet the needs of users at either synchrotron or XFEL light sources. The chips are designed to be customized around different types of crystals and offer users a friendly, quick, convenient, ultra-low-cost and robust sample-delivery platform. Compared with the previous iteration of the chip [Gilbile *et al.* (2021 ▸), *Lab Chip*, **21**, 4831–4845], the new design eliminates cleanroom fabrication. It has a larger imaging area to volume, while maintaining crystal hydration stability for both *in situ* crystallization or direct crystal slurry loading. Crystals of two model proteins, lysozyme and thaumatin, were used to validate the effectiveness of the design at both synchrotron (lysozyme and thaumatin) and XFEL (lysozyme only) facilities, yielding complete data sets with resolutions of 1.42, 1.48 and 1.70 Å, respectively. Overall, the improved chip design, ease of fabrication and high modifiability create a powerful, all-around sample-delivery tool that structural biologists can quickly adopt, especially in cases of limited sample volume and small, fragile crystals.

## Abstract 2

An improved design of an all-polymer microfluidic ‘chip’ for fixed-target serial crystallography is presented that can easily be fabricated in-house, is inexpensive and is highly modifiable to meet broad user needs for room-temperature serial crystallography at both synchrotron and XFEL light sources.

## 1. Introduction

X-ray crystallography has been the gold standard for protein structure determination. While data-collection limitations from large protein molecules are being eliminated by recent advances in cryoEM, X-ray crystallography techniques have been limited due to the need to grow large and well diffracting protein crystals. X-ray free-electron lasers (XFELs), light sources with a maxiumum brightness ten orders of magnitude higher than traditional synchrotron sources, offer a method to obtain protein structures from small and poorly diffracting crystal samples using the ‘diffract before destroy’ technique. Emerging serial crystallography techniques have correspondingly driven innovation in sample-delivery methods by creating the need to deliver up to millions of microcrystals at ambient temperature (Grünbein & Kovacs, 2019 ▸; Cheng, 2020 ▸). Several novel sample-delivery techniques have been developed to meet these requirements, with the most advanced being flow-based systems such as gas-focused dynamic virtual nozzle (GDVN) jets and high-viscosity extrusion, which are used to flow pre-loaded protein crystals past the X-ray beam interaction point (Echelmeier *et al.*, 2019 ▸; Martiel *et al.*, 2019 ▸). Alternatively, fixed-target systems, in which the crystals are fixed inside a sample holder which is rastered during data collection, have emerged as a viable alternative to flow-based target-delivery methods. Fixed-target sample delivery offers some significant advantages compared with other methods, including improved ease of use, reduced sample consumption, the ability to screen different sample conditions rapidly with potentially higher hit rates, and adaptability for varying experimental conditions and sample-geometry requirements (Echelmeier *et al.*, 2019 ▸; Ebrahim *et al.*, 2019 ▸; Horrell *et al.*, 2021 ▸).

Early fixed-target systems for XFEL serial crystallography were silicon-based chips with thin windows or pores that were fabricated using lithography or etching techniques and required crystal loading into specific window positions (Hunter *et al.*, 2014 ▸; Martiel *et al.*, 2021 ▸; Roedig *et al.*, 2016 ▸). These silicon-based chips offer ultralow-background data collection but require costly and difficult manufacturing processing and long lead times. Samples must also be freshly loaded immediately before measurements and cannot be stored. Polymer-based fixed-target systems such as nylon or polyimide loops, various polymer meshes and other fully polymer-based chips have been developed as an alternative (Feld *et al.*, 2015 ▸; Park *et al.*, 2020 ▸; Lee *et al.*, 2019 ▸, 2020 ▸). Although amorphous scattering from the polymer material and sample buffer increases the background scattering (Sui & Perry, 2017 ▸), polymer-based fixed-target systems are more accessible due to their low cost, ease of use and flexibility in geometry, fabrication and sample handling, including on-chip crystallization and sample storage (Huang *et al.*, 2020 ▸; Gicquel *et al.*, 2018 ▸; Gilbile *et al.*, 2021 ▸; Lyubimov *et al.*, 2015 ▸; Doak *et al.*, 2018 ▸). However, few designs can achieve ease of fabrication, enable *in situ* crystallization and slurry loading, and provide long-term stability in the same system.

Here, we describe a user-friendly, inexpensive, polymer-based microfluidic fixed-target system for protein crystal sample delivery for both serial femtosecond crystallography (SFX) and synchrotron serial crystallography (SSX) that dramatically improves on the design described in Gilbile *et al.* (2021 ▸). The original polymer chip was a robust, easy-to-use, low-background, fixed-target system and, importantly, was capable of *in situ* crystallization, with easy sample monitoring and stable, long-term storage. However, the fabrication process relied on lithography techniques, limiting its ease of production. As a result, changing the dimensions of the chip was very costly and time-consuming, limiting its adaptability and adoption by the broad structural biology community. Furthermore, the supporting framework limited the sample X-ray imaging area by 70%, obscuring many of the protein crystals in the sample layer. This work presents an improved design and methodology to address these problems, with a freestanding window area as large as 3.5 × ≥30 mm and minimal sample dead space. Additionally, the entire chip can be realistically designed, fabricated, assembled and loaded within a single day using widely accessible equipment, and is also compatible with standard magnetic bases for goniometer mounting. The simplification of the design enables rapid modifiability, including changes in chip dimensions, imaging area shape, loading techniques and even material, making it possible to tailor the new fixed-target chip for various beamline requirements, experiments or protein crystal types.

## 2. Materials and methods

Cyclic olefin copolymer (COC; TOPAS Advanced Polymers Grade 6017, *T*_g_ = 170°C) was purchased from Polysciences Inc., USA. Polyvinyl alcohol (PVA) was purchased from Sigma–Aldrich (Product 363170). *Sec*-butylbenzene (99.0%, TCI America, catalogue No. B0714500ML) was used as the solvent to dissolve the COC. As a supporting frame material, 1 mm thick transparent polymethyl methacrylate (PMMA) sheets (SimbaLux, 5′′ × 7′′) were purchased from Amazon. The double-sided acrylic adhesive was purchased from Adhesives Research (ARcare 92712) and adhesive transfer tape was purchased from 3M (F9460PC). Chicken egg-white lysozyme (catalogue No. L6876) and thaumatin (catalogue No. T7638) were purchased from Millipore Sigma (St Louis, Missouri, USA).

### 2.1. Chip fabrication and assembly

**Figure 1:**
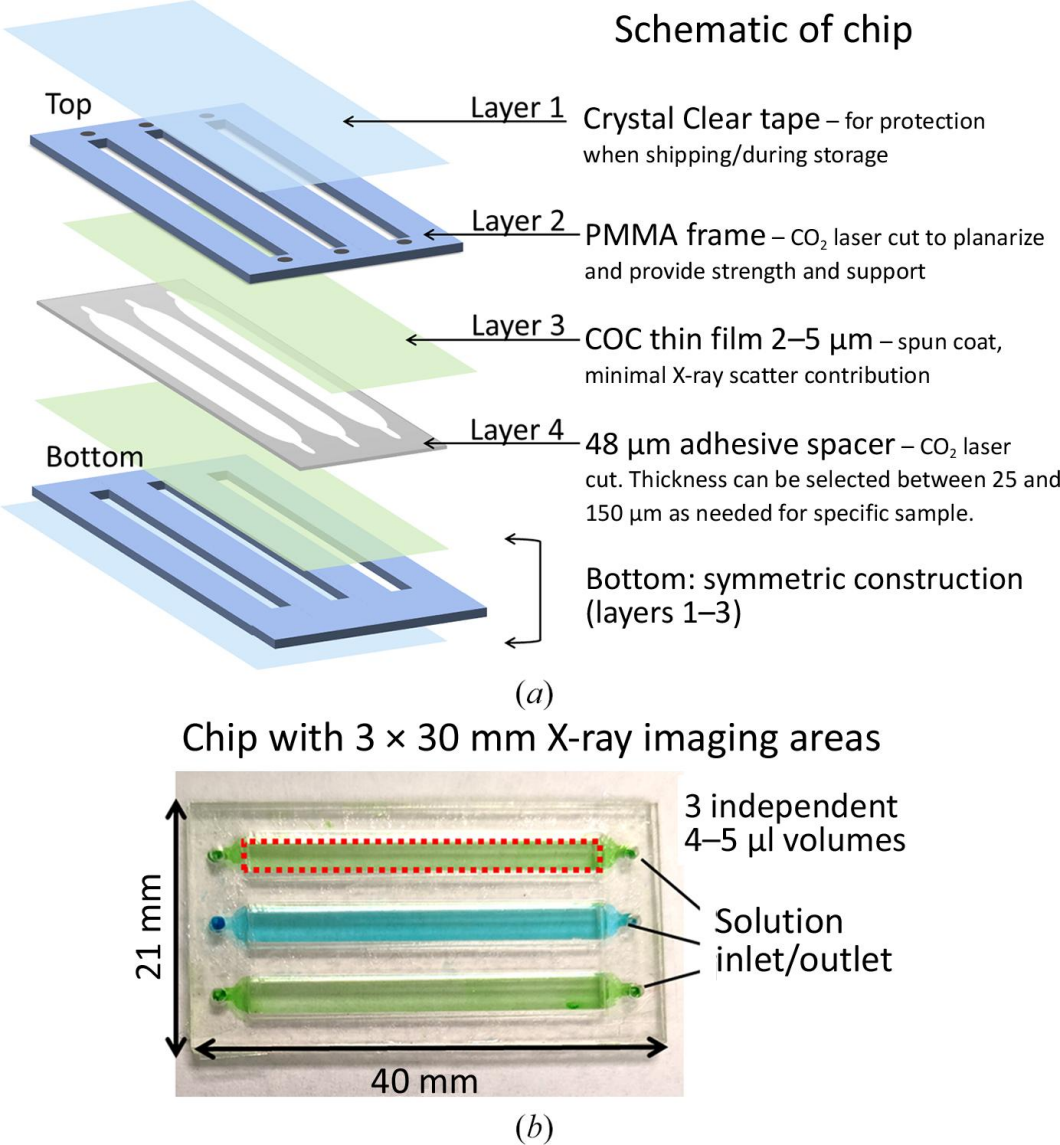
(*a*) A schematic of the improved chip construction layers. Note that ‘top’ frames (layer 2) have inlet holes while ‘bottom’ frames do not. (*b*) Image of the assembled chip with sample X-ray imaging areas filled with colored solution. The dashed red line demarks the active X-ray imaging area. The sample is loaded by manual pipetting into one of the inlet/outlet holes. Each sample-imaging window is independent.

**Figure 2:**
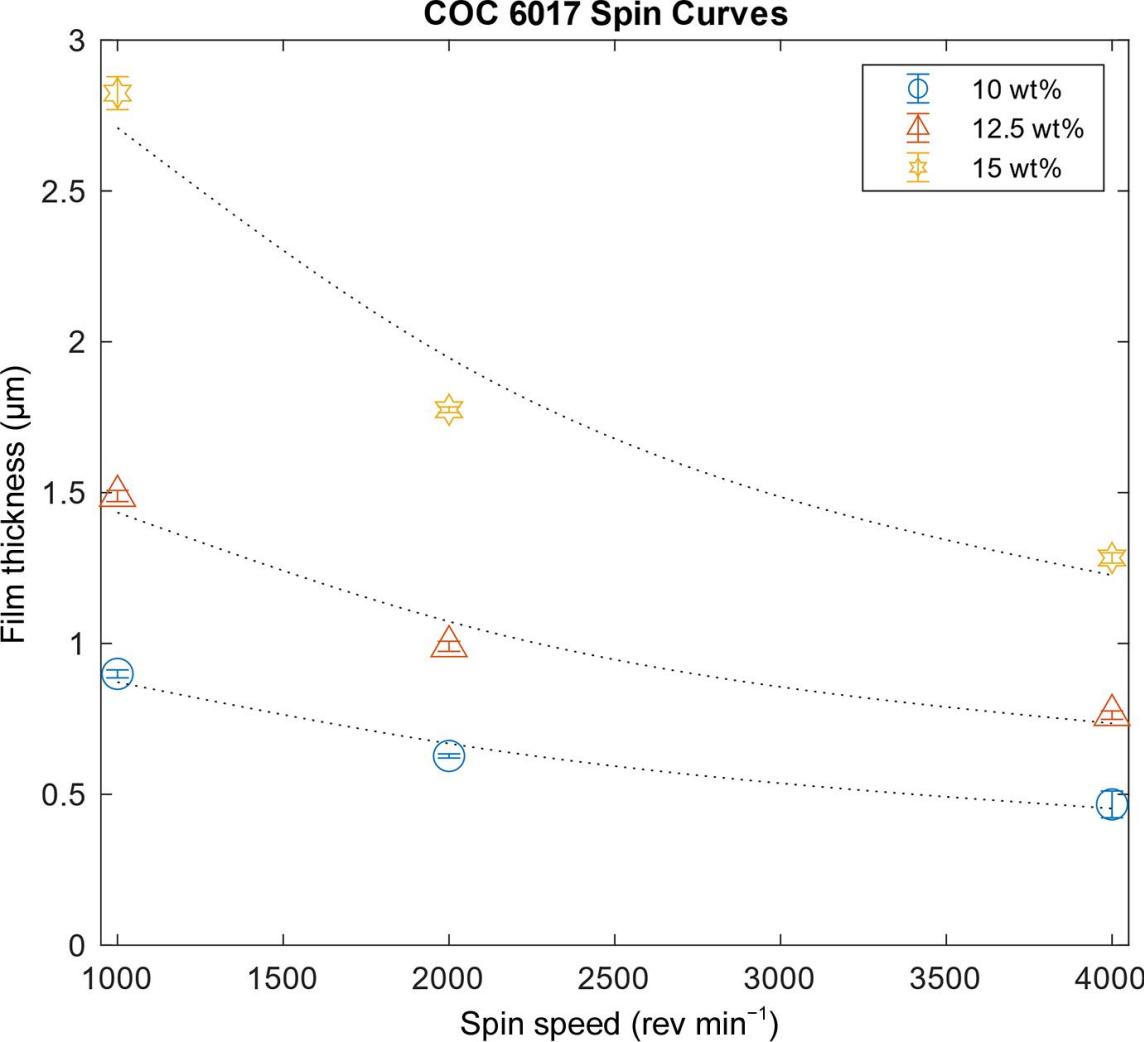
Spin curves showing COC film thickness as a function of spin speeds for different concentrations of COC 6017 dissolved in *sec*-butylbenzene.

A schematic of the four different layers and assembly of the microfluidic chip is shown in Fig. 1 ▸(*a*). The enclosed X-ray imaging areas are made of thin-film COC (layer 3) prepared by spin-coating solutions of COC onto silicon wafers. The solutions were prepared by dissolving 15 wt% COC in *sec*-butylbenzene at 120°C overnight or until fully dissolved. Varying film thicknesses from 500 nm to 5 µm are possible depending on the spin speed and solution concentration. Fig. 2 ▸ shows spin curves for 6017 COC. While thicker films provide better support and lower water permeability, users should select the smallest possible film thickness to ensure a low background. To improve the delamination of the COC thin film from the silicon wafer, a water-soluble sacrificial layer of 9 wt% PVA in Milli-Q water was first spun onto a clean, UV–ozone-treated silicon wafer before COC film deposition. A 15 min UV–ozone treatment was used to improve the surface wettability of the silicon wafer. The PVA sacrificial layer was baked on a hotplate at 120°C for a few minutes to fully evaporate residual water. Afterwards, warm COC solution (>80°C) was spun on top of the dried PVA layer at 1000 rev min^−1^ for 60 s.

Supporting frames (layer 2) were constructed from 1 mm PMMA with polyester adhesive transfer tape applied on one side of the PMMA sheet before cutting. The PMMA with polyester laminate was CO_2_ laser-cut; the cutout areas define the sample-imaging window. A CO_2_ laser was also used to cut the 48 µm double-sided acrylic adhesive spacer (layer 4). For a single flow channel with 2.7 µm 6017 COC film and 48 µm spacer, we found that a width of 3.5 mm is close to the maximum, with larger widths sometimes resulting in thin-film contacts across the air gap before sample loading. These dimensions are widely compatible with most crystallization solution viscosities and crystal slurries with a largest dimension of less than 30 µm. For different beamline needs, the dimensions of both the overall chip size and imaging windows are easily customizable. For an improved signal-to-noise ratio, the thickness of the sample flow layer should be matched to the crystal size to minimize the background from the crystallization solution. Adhesive spacers from 25 to 150 µm are commercially available.

The PMMA frame (layer 2) was placed onto the COC film (layer 3) with the polyester adhesive side down on the spun-coated COC film to assemble the chip. By pressing firmly on all parts of the PMMA frame, strong adhesion is formed between the supporting frame and the COC film. Multiple frames can be pressed on a single wafer. Afterwards, the adhered assembly was soaked in Milli-Q water until the frames with attached COC film were delaminated from the wafer. This process takes up to 36 h, but can be accelerated by using a razor blade to cut around the outer edges of the PMMA frame before soaking. The delaminated chip halves were then rinsed with Milli-Q water and dried using a gentle stream of N_2_ gas. Only one of the PMMA frames has inlet/outlet holes (Fig. 1 ▸ *a*, top versus bottom). An adhesive spacer (layer 4) was aligned and placed onto one of the frame-supported COC windows to complete the construction. A second completed half (layers 2 and 3) was then placed on the spacer, forming the chip (Fig. 1 ▸ *b*). Detailed fabrication steps and vector files for laser cutting can be found in the supporting information.

### 2.2. Sample loading

The chips are compatible with direct crystal slurry loading and *in situ*, on-chip crystallization (micro-batch or vapor-diffusion conditions) by directly pipetting either crystal slurry or protein/precipitant solution into an inlet hole (Fig. 1 ▸ *b*). Sample solution flows into the imaging channels through capillary action (Sui *et al.*, 2021 ▸). Once samples are loaded, Crystal Clear tape (layer 1) can be wrapped around the chip to reduce water loss during batch crystallization to improve sample stability or it can be left unwrapped for vapor-diffusion conditions. In both cases, *in situ* crystallization times are typically longer due to the high aspect ratio of the sample volume and the limited area for evaporation.

Lysozyme and thaumatin were used as model proteins to demonstrate chip performance. Commercially available lyophilized samples of each protein were dissolved in Milli-Q water to produce protein solutions of 50 mg ml^−1^ lysozyme and 25 mg ml^−1^ thaumatin. Stock solutions of 2 *M* NaCl with 0.1 *M* sodium acetate buffer pH 4.6 and 1 *M* l-sodium potassium tartrate with 0.1 *M* ADA buffer pH 6–6.5 were used as the precipitant solutions for lysozyme and thaumatin, respectively. For lysozyme, we also explored the use of hydrogels during crystallization to further demonstrate the utility of the chip for different crystallization conditions. In this case, 2 wt% low-melting-point agarose (purchased from Hampton Research) was heated to 85°C, cooled to 40°C and then added to the precipitant solution and mixed with the protein solution. The final mixture with 0.3 wt% agarose was pipetted above 30°C into an inlet of the chip until the channel was filled. The total sample volume in each lane is 4 µl. For synchrotron measurements, larger protein crystals were desired and were crystallized *in situ* under micro-batch conditions and typically sealed with Crystal Clear tape to improve hydration stability. Supplementary Fig. S1 shows optical microscopy images of lysozyme crystals grown *in situ* inside a chip that was kept under ambient conditions. Over ten days, no significant dehydration was observed under ambient conditions. The crystals showed no directional preference when grown *in situ*.

### 2.3. Protein diffraction measurements

**Figure 3:**
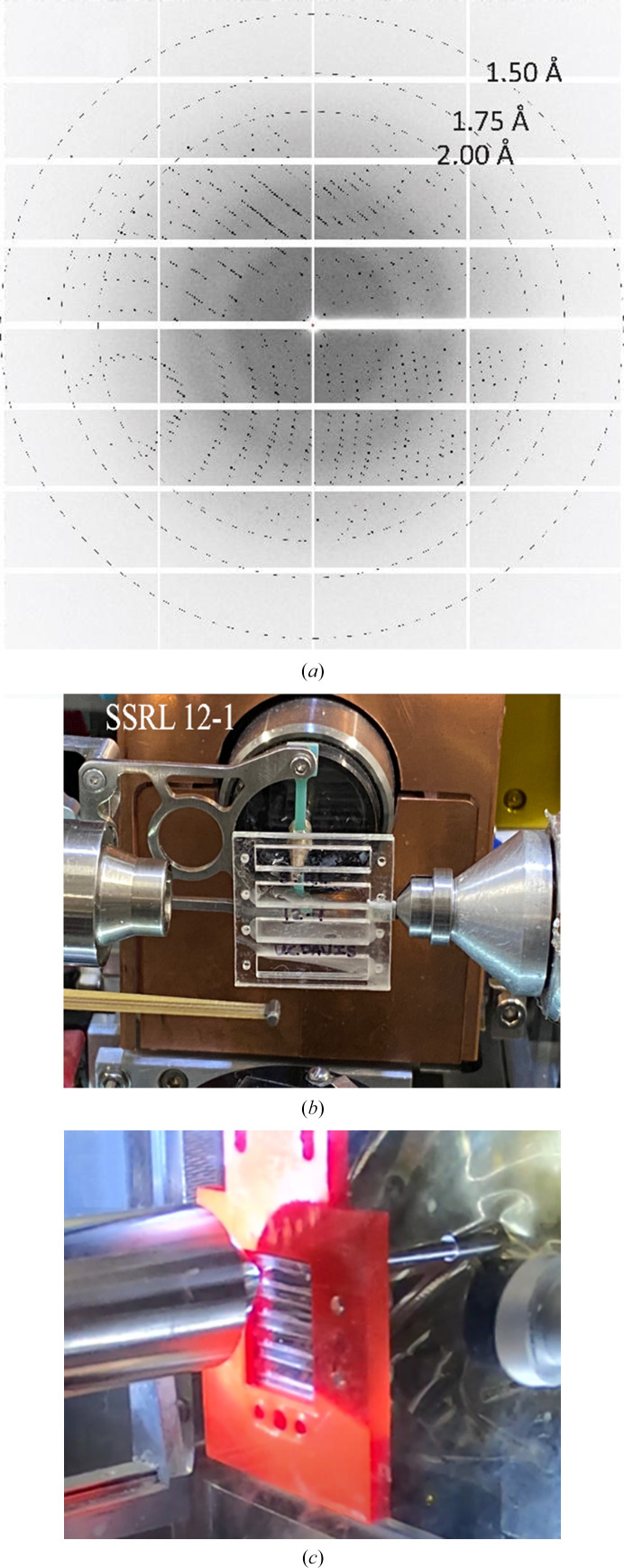
(*a*) X-ray diffraction pattern obtained from a thaumatin crystal at 10% transmission at SSRL. A 1′′ × 1′′ chip design with four independent volumes is mounted on (*b*) beamline 12-1 at SSRL and (*c*) the MFX beamline at LCLS (in a red 3D chip holder).

Two model proteins, lysozyme and thaumatin, were crystallized on a chip and measured at ambient temperature on beamline 12-1 at the Stanford Synchrotron Radiation Lightsource (SSRL). A screw-tightened, slotted holder with a magnetic base was used to hold the PMMA frame portion of the chip during diffraction experiments (Fig. 3 ▸ *b*). The magnetic base was mounted onto the goniometer at the beamline. Diffraction data were collected at a wavelength of 0.9794 Å with a beam size of 0.05 × 0.04 mm using an EIGER X 16M detector (Dectris AG) at a detector distance of 0.2 m. The beamline sample-holder translational motors were used to align and center individual single crystals to the beam path, using inline high-resolution cameras to identify each crystal. Data sets were collected from these centered single crystals over 40° wedges. Diffraction data from 15 and 14 individual crystals (50 µm in diameter on average) were merged to give complete data sets for lysozyme and thaumatin, respectively. An exemplar diffraction pattern from thaumatin using the improved chip is shown in Fig. 3 ▸(*a*).

Lysozyme crystals were grown *in situ* and measured on the Macromolecular Femtosecond Crystallography (MFX) beamline at the Linac Coherent Light Source (LCLS). A different crystallization protocol was applied to produce smaller (10 µm on average) dispersed lysozyme crystals for comparative XFEL measurements (50 mg ml^−1^ lysozyme with a 1:1 ratio of protein solution to mother liquor: 1 *M* NaCl, 0.1 *M* sodium acetate pH 4.6). XFEL diffraction data were collected at ambient temperature in a helium-rich ambient (HERA) environment to reduce background from air scattering. A 1′′ × 1′′ chip with four 3.5 × 18 mm channels was fabricated to match the MFX sample-stage displacement range. A 3D-printed chip holder was made to attach the chip to the sample stage (Fig. 3 ▸ *c*). Diffraction data were collected at 1.253 Å and 11% transmission using the SLAC ePix10k2M detector. The beamline sample holder translational motors were used to align and continuously raster the chip at 120 Hz. Variable shot spacings between 25 and 200 µm were possible at 120 Hz depending on the linear translational motor speed, and data collection was primarily at 50 µm displacements. The inline camera resolution was granular, so larger raster scans were used to optimize the data-collection efficiency to ensure that the sample window area was fully imaged.

## 3. Results and discussion

### 3.1. Improved chip fabrication and assembly

As detailed in Table 1 ▸, the new chip generation (Chip 2.0) provides significant advancements in manufacturing time and cost, along with a larger continuous imaging area and reduced sample volume:area ratio. More shots per chip are possible by having a large, continuous window area, maximizing sample-to-data efficiency. The ease of fabrication and improvements in turnaround times indicate that chips can be mass fabricated and quickly adjusted for specific beamline requirements. Users can easily make their own chips based on their specific needs, requiring only a spin coater and a CO_2_ laser cutter. The ability to optimize chips to match specific crystal types and measurement requirements enhances the chip versatility and its applicability for a range of experiment types.

### 3.2. Microfluidic chip background scattering

**Figure 4:**
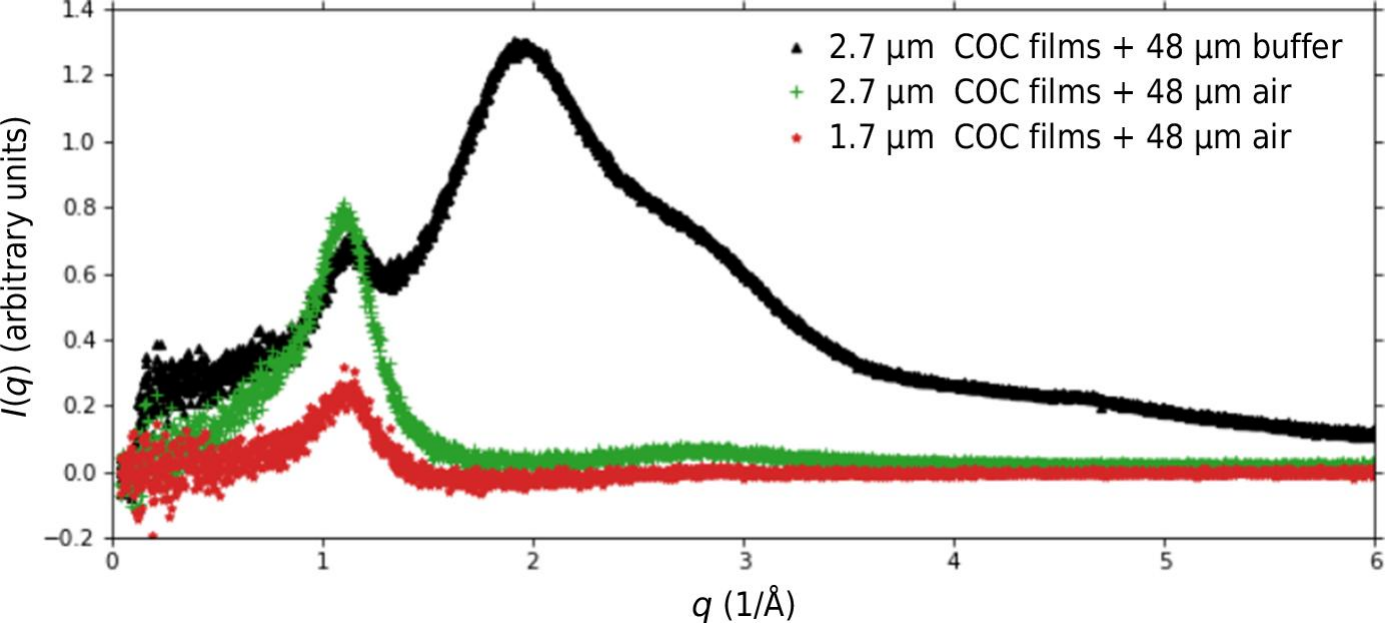
Radial X-ray scattering background with air scattering subtracted measured on beamline 12-1 at SSRL. The water solvent ring at *q* = ∼1.8 Å^−1^ is evident in the filled chip (black data points). While the background scatter from the COC windows at *q* = ∼1.2 Å^−1^ is effectively controllable based on the film thickness (green versus red data points), it may exhibit minor fluctuations (green versus black data points) due to small variations in the film thickness across the film and among different chips.

Fig. 4 ▸ presents the background radial scattering from two chips with different COC window thicknesses and a 48 µm spacer on beamline 12-1 at SSRL. Background scattering was measured for empty and buffer-filled chips. The majority of the background scatter from the chip was from the aqueous buffer, with a small diffuse peak from the COC films at approximately *q* = 1.2 Å^−1^, consistent with previous studies (Martiel *et al.*, 2021 ▸; Ren *et al.*, 2018 ▸; Gilbile *et al.*, 2021 ▸). For the chip demonstration measurements, the same channel geometries were used for consistency. The smaller crystals used at XFELs therefore have a relatively thicker layer of buffer surrounding them, resulting in a higher background. The chip can be further optimized and tailored to different beamline needs. Users are encouraged to choose the smallest channel thickness for an optimized signal-to-noise ratio. Conversely, higher intensity experiments can be paired with a slightly thicker window and more support structures.

Comparing the background scattering from the two different COC window thicknesses, a decrease in the COC thickness from 2.7 to 1.7 µm decreased the peak COC scattering contribution by approximately a factor of four. Overall, using a dramatically reduced COC film thickness (1–5 µm) contributes much less background scatter compared with similar systems based on thick (600–700 µm) COC sheets (Pinker *et al.*, 2013 ▸; Vasireddi *et al.*, 2022 ▸).

### 3.3. Protein crystal diffraction

#### 3.3.1. Synchrotron measurements

High-resolution diffraction data sets were collected at room temperature. For lysozyme the structure was refined to *R*_work_ and *R*_free_ values of 0.185 and 0.212, respectively, at a resolution of 1.45 Å. Furthermore, the structure of thaumatin was refined to *R*_work_ and *R*_free_ values of 0.139 and 0.153, respectively, at a resolution of 1.48 Å. The thaumatin structure resolution achieved is comparable to those from other fabricated and commercially available fixed-target systems grown and loaded under similar conditions at synchrotron sources (PDB entries 3zej, 5a47 and 6xbx; Pinker *et al.*, 2013 ▸; Zander *et al.*, 2015 ▸; Gavira *et al.*, 2020 ▸). The lysozyme and thaumatin synchrotron data have been deposited in the PDB (as PDB entries 8scy and 8fzw). Compared with the previous generation of chips (Gilbile *et al.*, 2021 ▸), the diffraction resolution for lysozyme was improved in 0.3 wt% agarose solution.

One point to emphasize is that a single chip was sufficient for data collection, requiring only 0.6 and 0.3 mg of lysozyme and thaumatin, respectively. In addition to lysozyme and thaumatin, diffraction data from slurry-loaded, wild-type Nsp15 endoribonuclease (NendoU; Jernigan *et al.*, 2023 ▸) was collected using the chip. These measurements demonstrate that a range of samples and conditions can be efficiently run during a single measurement period. Additionally, the lysozyme and thaumatin samples were crystallized *in situ* days in advance. We have maintained samples on chips for weeks with no observable decrease in sample quality. Optical microscopy can be used to pre-select optimal chips and sections of chips before beamtime, significantly increasing operational efficiency and dramatically reducing user stress during beamtime.

#### 3.3.2. XFEL measurements

**Figure 5:**
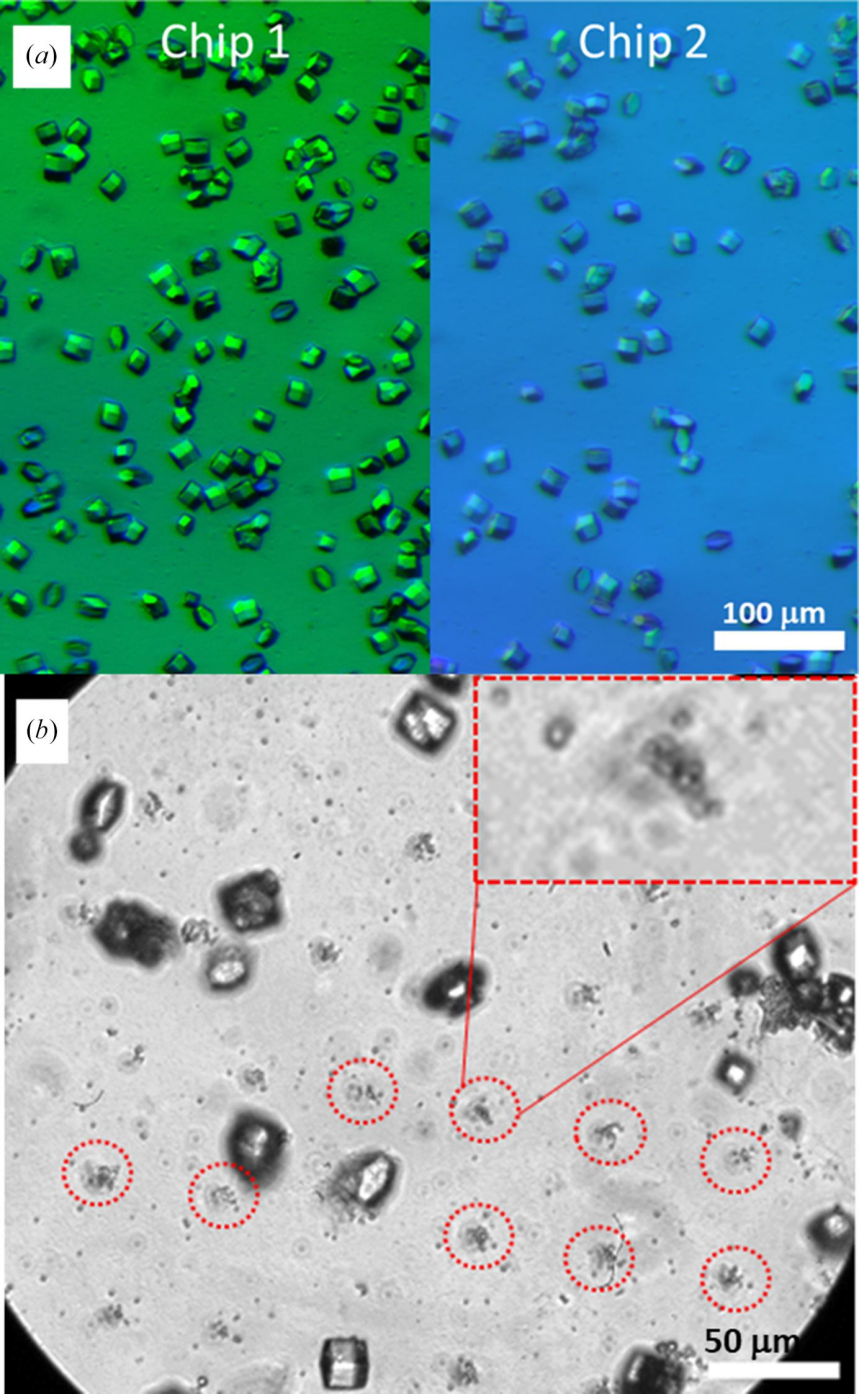
(*a*) Representative images of lysozyme crystals and the variation in crystal density in the two chips before XFEL measurements (the scale bar applies to both parts of the figure). (*b*) Lysozyme crystals five days after XFEL measurements at 50 µm shot spacing, 11% transmission and 0.9–1.0 mJ pulse energy. Small vapor bubbles of a few micrometres in size were observed in some areas of the microfluidic channel where the beam was rastered, as highlighted by the dashed circles and the inset.

**Figure 6:**
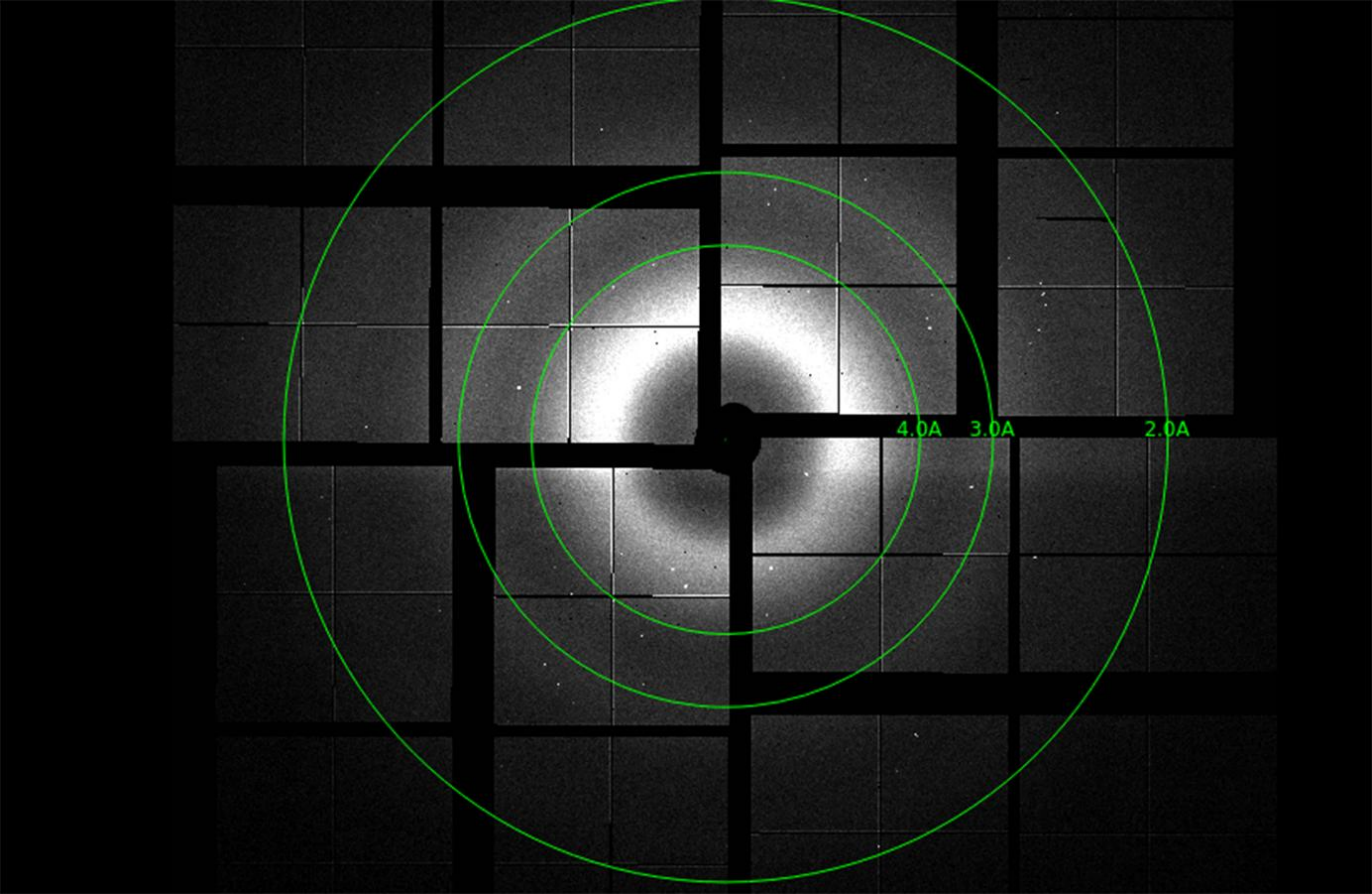
X-ray diffraction pattern obtained from a lysozyme crystal at 11% transmission on the MFX beamline at LCLS. Raw figure without any background subtraction.

To better represent XFEL samples, conditions that encouraged nucleation over crystal growth using *in situ* crystallization were selected. Microscopy images of the two chips used for data collection are shown in Fig. 5 ▸(*a*). Diffraction from small, dispersed lysozyme crystals of about 10–15 µm in the largest dimension with randomized orientations was obtained. An exemplar diffraction pattern is shown in Fig. 6 ▸ without any image processing. The merging statistics obtained from the data sets collected are shown in Table 2 ▸.

**Figure 7:**
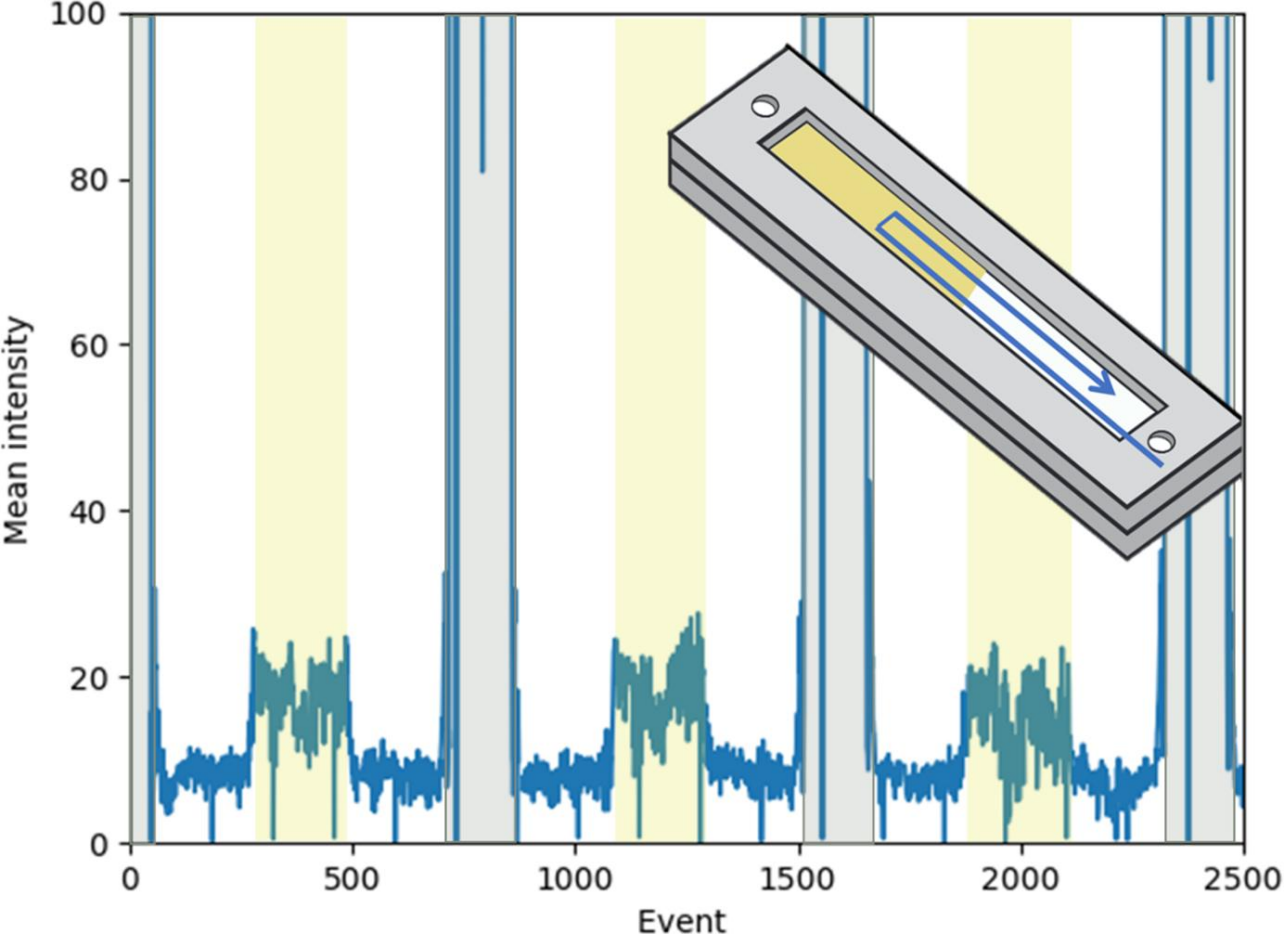
Mean detector intensity plot per XFEL shot while rastering a half-filled chip. The gray areas are where PMMA frames are scanned, the yellow areas are samples (buffer and diffracting crystals) and the blank areas are polymer chips with air only. X-ray shots that hit the PMMA frame are easily excluded from analysis using a mean intensity cutoff. The intensity from the samples was roughly three times the blank air background.

For these first XFEL demonstration measurements, the transmission was 11% and the full repetition rate of 120 Hz was used. The inline camera image quality was relatively poor. Instead of carefully aligning the imaging X-ray window for the raster scan, the chips were crudely aligned and rastered over a wider area to ensure that the entire sample window was measured. Because the chips are entirely polymer and sample, there is no issue with rastering the frame or any region of the chip through the X-ray beam, eliminating the need for careful positioning or precision rastering. X-ray shots on the thick PMMA supporting frames have a much higher mean detector intensity and are easily excluded with an intensity cutoff when analyzing the data, as shown in Fig. 7 ▸. In this case, we ran a partially filled lysozyme chip as well to provide a rough estimate of the background of the chip relative to the sample.

Using a wide scan area, data collection from two chips took about 1 h, yielding 47 948 hits, from which 29 215 lattices were indexed. Two imaging windows were re-run to exhaust the remaining crystals, and the total indexed hit rate was 13.5%. Data were merged and refined to *R*_work_ and *R*_free_ values of 0.2512 and 0.2814, respectively, at a resolution of 1.70 Å. Fig. 5 ▸(*b*) shows an image of one of the chips after X-ray rastering. No damage to or significant dehydration of the microfluidic channel was observed, even after multiple scans. The chip was imaged five days after XFEL measurements, demonstrating the robustness of the chip and stability against dehydration. With the higher magnification available at this time, it was possible to detect patterns of tiny vapor bubbles with a 50 µm pitch in some regions, presumably adhered to the hydrophobic COC window film, as well as some vapor bubbles that diffused. Notably, the COC film and chip were still fully intact.

**Figure 8:**
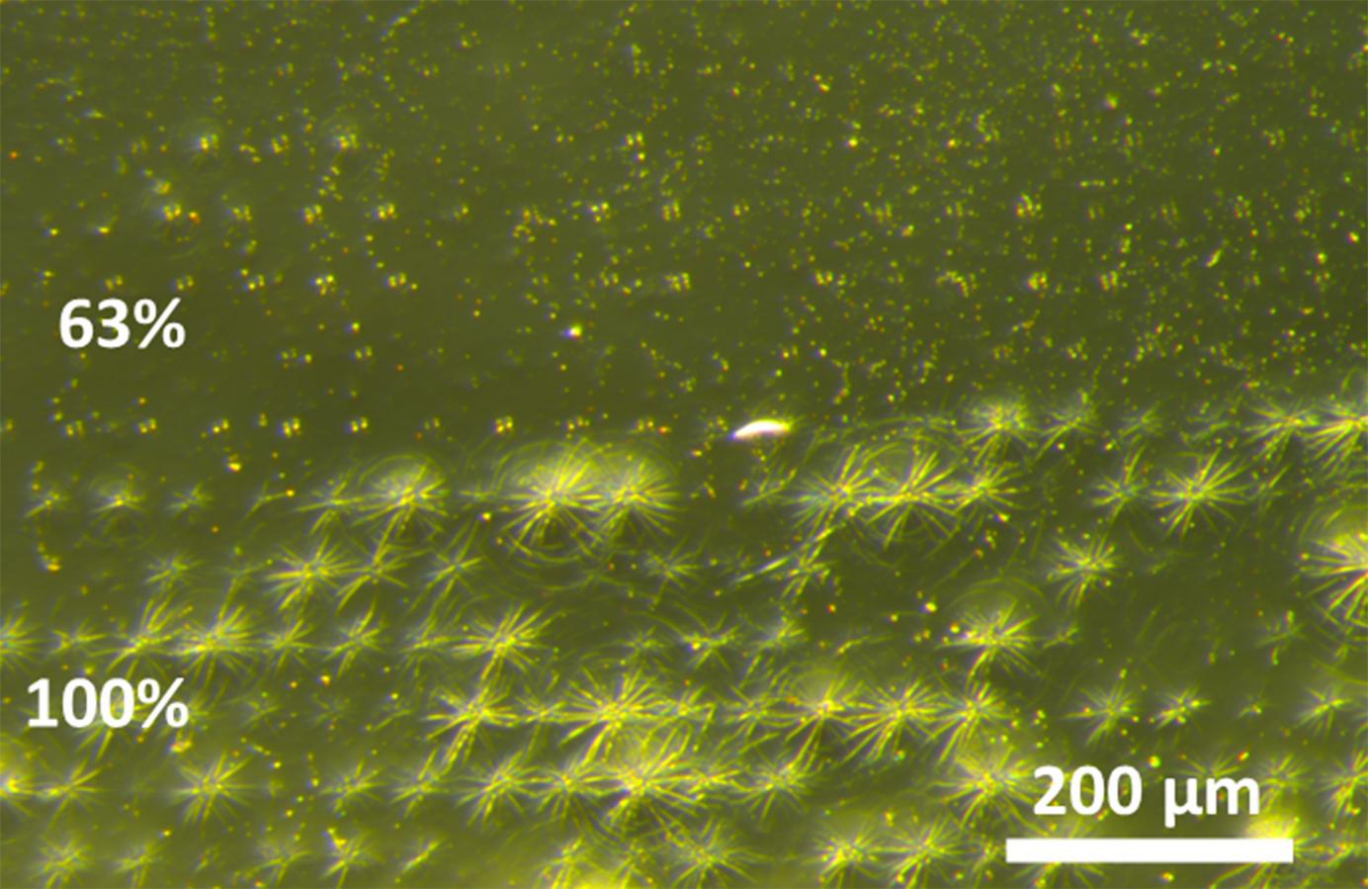
Chip after 63% (top) and 100% (bottom) transmission. The 50 µm shot spacing is clearly visible in both cases, but no change in hit rate or dehydration was found at 63% transmission. At 100% transmission dehydration and diffraction from salt crystals were found, demonstrating that the X-ray shots likely led to cracks in the COC film or pinhole defects.

An additional chip with different protein screening samples was run at a range of transmissions. No decrease in hit rates was observed with up to 63% transmission. However, at 100% transmission diffraction from salt crystals was observed, which progressively increased over time. Dehydration was visible when the chip was removed from the beamline. Fig. 8 ▸ shows an optical image of a chip during the transition from 63% to 100% transmission. The 50 µm shot-spacing pattern is clearly evident, and a dramatic pattern in the COC film is observed at 100% transmission. Subsequent imaging at four times higher magnification did not detect perforations in the COC film. However, the film must have minor defects along the striated pattern, resulting in sample dehydration during measurement. Future work will carefully study the chips at high transmission to optimize for these conditions. In particular, the use of a 25 µm adhesive layer thickness will dramatically decrease the buffer background and water adsorption, while a high-humidity helium chamber may allow full operation without any changes by preventing sample dehydration.

## 4. Conclusions

Highly efficient, low-sample-consumption and easy-to-use sample-delivery methods are crucial to maximize the potential of SFX and SSX techniques for serial crystallo­graphy. This paper presents a methodology for fabricating an inexpensive, low-background and highly versatile design for fixed-target SFX and SSX sample delivery without needing lithography or etching steps. Since chip fabrication requires only a spin coater and a CO_2_ laser cutter, our approach makes customizable fixed-target devices available to a broader community. The chips are compatible with different sample-loading modalities, including crystal slurry loading, micro-batch crystallization and vapor-diffusion crystallization. Diffraction data from two model proteins crystallized on-chip demonstrate that high-quality diffraction data can be obtained at ambient temperature using our device at both synchrotron and XFEL light sources. In addition, the ability to rapidly reconfigure the chip geometry and dimensions allows the user to customize the chip to match their specific sample, for example by (i) tailoring the spacer thickness to match the crystal size and reduce background scattering from the buffer, (ii) changing the film thicknesses or the grade of COC for different water-loss profiles, (iii) changing the imaging window dimensions to match sample-volume limitations and (iv) altering the overall chip size and shape for unique beamline fixed-target mounting configurations.

Compared with the previous-generation device reported in Gilbile *et al.* (2021 ▸), the new chip design and improved fabrication method offer comparable *in situ* crystallization conditions, crystal slurry loading and sample stability, while providing a dramatic improvement in the ease of fabrication, an increase in X-ray imaging window size with a concomitant decrease in dead volume, and rapid modifiability. Looking forward, many alterations/additions are currently being developed, including (i) removing excess buffer before data collection to reduce background contributions, (ii) controlling crystal nucleation density using electric fields (Alexander & Radacsi, 2019 ▸), (iii) controlling crystal nucleation locations using polymer brushes on the COC imaging window, (iv) design modifications for use with membrane proteins in the highly viscous lipidic cubic phase and (v) maintaining sample stability for measurements under vacuum.

## Supplementary Material

PDB reference: thaumatin, 8fzw

PDB reference: lysozyme, 8scy

PDB reference: 8sil

Supporting information including Supplementary Figures and Table. DOI: 10.1107/S2059798323007027/wa5145sup1.pdf

## Figures and Tables

**Table 1:** Advancement in the new generation, Chip 2.0, compared with the previous generation, Chip 1.0

	Previous design (Chip 1.0)	New design (Chip 2.0)
Thin-film material	COC TOPAS Advanced Polymers Grade 8007, high heat deflection	COC TOPAS Advanced Polymers Grade 6017, improved mechanical properties for a larger freestanding window area
Fabrication needs	Cleanroom microfabrication equipment required	Easily accessible CO_2_ laser cutter and spin-coater only
Design modification time	Weeks (microfabricated silicon mask)	<1 day
X-ray imaging area (mm^2^)	43	90 (easily modified)
XFEL shots per chip at 50 µm shot spacing	17200	100800 for 1′′ × 1′′ chip (4 × 3.5 mm × 18 mm sample-imaging areas)
Imaging area-to-volume ratio (mm^2^ µl^−1^)	5.4	18
Features	Only demonstrated with *in situ* crystallization	Slurry loading and *in situ* crystallization

**Table 2:** Crystallographic statistics obtained for lysozyme and thaumatin Values in parentheses are for the highest resolution shell.

Protein	Thaumatin	Lysozyme (synchrotron)	Lysozyme (XFEL)
Average crystal diameter (µm)	∼50	∼50	∼10
Resolution range (Å)	36.39–1.48 (1.53–1.48)	39.57–1.45 (1.47–1.45)	24.82–1.70 (1.76–1.70)
*a*, *b*, *c* (Å)	58.67, 58.67, 151.56	79.15, 79.15, 38.05	78.80 ± 0.3, 78.80 ± 0.3, 38.0 ± 0.2
α, β, γ (°)	90, 90, 90	90, 90, 90	90, 90, 90
Space group	*P*4_1_2_1_2	*P*4_3_2_1_2	*P*4_3_2_1_2
Total reflections	463922 (46703)	291020 (10061)	22394 (13653)
Unique reflections	45060 (4434)	22046 (1243)	13193 (1353)
Multiplicity	10.3 (10.5)	13.2 (13.1)	809 (435)
Completeness (%)	99.87 (99.95)	100.0 (99.9)	99.8 (100)
〈*I*/σ(*I*)〉	10.89 (0.95)	12.6 (1.03)	4.6 (0.5)
CC_1/2_	0.998 (0.572)	0.999 (0.999)	0.9480 (0.4542)
Wilson *B* factor (Å^2^)	19.67	21.77	15.83
