# Supplementary material for: A user-friendly plug-and-play cyclic olefin copolymer-based microfluidic chip for room-temperature, fixed-target serial crystallography
Source: Acta Crystallogr D Struct Biol. 2023 Sep 25;79(Pt 10):944–52. doi: 10.1107/S2059798323007027 (PMC10565732; doi:10.1107/S2059798323007027)
Supplement: Supplementary file 1 [file d-79-00944-sup1.pdf]

STRUCTURAL
BIOLOGY

Volume 79 (2023)

Supporting information for article:

A user-friendly plug-and-play cyclic olefin copolymer-based microfluidic chip for room-temperature, fixed-target serial crystallography

Zhongrui Liu, Kevin Gu, Megan L. Shelby, Deepshika Gilbile, Artem Y. Lyubimov, Silvia Russi, Aina E. Cohen, Sankar Raju Narayanasamy, Sabine Botha, Christopher Kupitz, Raymond G. Sierra, Fredric Poitevin, Antonio Gilardi, Stella Lisova, Matthew A. Coleman, Frank Matthias and Tonya Kuhl

S1. Detailed protocol for fabricating new generation cyclic olefin copolymer (COC) microfluidic chips

S1.1. Laser cutting – PMMA frame and adhesive spacer

First, a double-sided pressure-sensitive adhesive tape (3M F9460PC) was applied to 1mm thick polymethyl methacrylate sheets (PMMA, Simbalux Acrylic Sheet, 5'x7'). Ethanol combined with a gentle N_2 stream was used to clean the surface of the PMMA frame to remove dust particles before applying the adhesive tape. Slow application of the adhesive is necessary to prevent trapping air. A Trotec Speedy 400 CO_2 laser cutter was used to cut the rigid PMMA frames and X-ray imaging windows. For one chip, two designs need to be made. A 'top' frame should include two roughly 1mm diameter circular inlet/outlet holes displaced approximately 2mm from the edge of each imaging window, while a 'bottom' frame does not have inlet/outlet holes. Similarly, a 25-µm or 48-µm spacer film (AR 92734 and AR92712, respectively, Adhesives Research Inc., Glen Rock, PA, USA) was laser cut to make the sample flow layer. The sample flow layer should extend to the outermost point of the inlet holes cut in the 'top' PMMA frame so that there is a channel between the inlet hole and the sample imaging window to allow for sample loading and air to escape.

S1.2. Spin-coating solutions

Polyvinyl alcohol (PVA) flakes/powder were dissolved in MilliQ water at 80°C on a hot plate with a magnetic stir bar until fully dissolved to make a 9 wt.% solution. While still warm, the PVA solution was filtered through a 0.22 µm PES membrane filter (Millex-GP, Millipore) to remove large aggregates or contaminant particles.

Cyclic olefin copolymer (COC) was dissolved in sec-butylbenzene at 120 °C on a hot plate with a magnetic stir bar until dissolved to produce a 15 wt% solution. While still warm, the COC solution was filtered through a 5 µm PDVF membrane filter (Millex-SV, Millipore). At room temperature, the COC solution solidifies, so the solution needs to be reheated and refiltered before spin coating.

S1.3. Thin film fabrication and spin curves

Silicon wafers were used as the substrates for COC film fabrication. Before use, the silicon wafers were cleaned in acetone, isopropanol, and water. Cleaned wafers were then UV-ozone (Jelight UVOX Model No.342) treated for 15 minutes to hydroxylate the wafer to improve surface wettability.

The PVA solution was spun coat onto the treated silicon wafer at 1000 rpm for 1 minute. The specific thickness of this layer is relatively unimportant as the layer is dissolved during the delamination process. However, the PVA layer should be spun carefully to maintain uniformity, as the film uniformity will

affect the subsequent COC film deposition. After spin-coating, the PVA film was baked on a hot plate at 130 °C for 10 minutes to ensure complete solvent evaporation.

A COC solution was then spun-coat on top of the PVA sacrificial layer at 1000 rpm for 1 minute. After spin coating, the COC film was baked on a hot plate at 130 °C for 10 minutes to ensure complete evaporation of the solvent.

Actions should be taken to minimize dust accumulation on the wafer surface and spun films. Any particles will affect the film quality. Surface uniformity can be judged by a uniform surface coloration and quantified through profilometry or ellipsometry measurements. To minimize particle contamination, all surfaces and spin-coating were done in class 100, horizontal Laminar flow cabinet (Labconco Purifier Clean Bench).

S1.4. Assembly

To attach the COC thin window film onto the PMMA frame, the PMMA frames were adhered to the spun-coated COC film while still on the silicon wafer. The PMMA frame was manually placed onto the wafer and pressed firmly by hand on all parts of the frame to ensure complete adhesive coverage between the PMMA frame and the underlying COC film. A razor blade was used to cut around the edges of the PMMA frame to help water penetrate the PVA sacrificial layer underneath the adhered frame. The entire wafer was soaked in MilliQ water until the frame & film (half-chip) delaminated from the wafer. Note that multiple frames can be placed on a single wafer to maximize efficiency. The half-chip was dried using a gentle stream of N_2 gas and can be surface hydrophilized through UV-ozone or plasma treatment before whole chip assembly. However, this step was unnecessary or used in the studies reported here. To complete a chip, the top and bottom half-chips were connected using the pressure-sensitive adhesive flow layer by manually aligning and bonding the adhesive onto both the PMMA frames. Placing a metal plate (load) overnight on the chips was also found to improve sealing by increasing the uniformity of the adhesive sealing between the spacer layer and the COC film.

S2. Chip Performance

S2.1. In-situ crystal growth and stability

Figure S1 shows optical microscopy images of lysozyme crystals grown in-situ inside the chip that was kept at ambient conditions. Over ten days, no significant dehydration was observed under ambient conditions. The crystals showed no directional preference when grown in-situ.

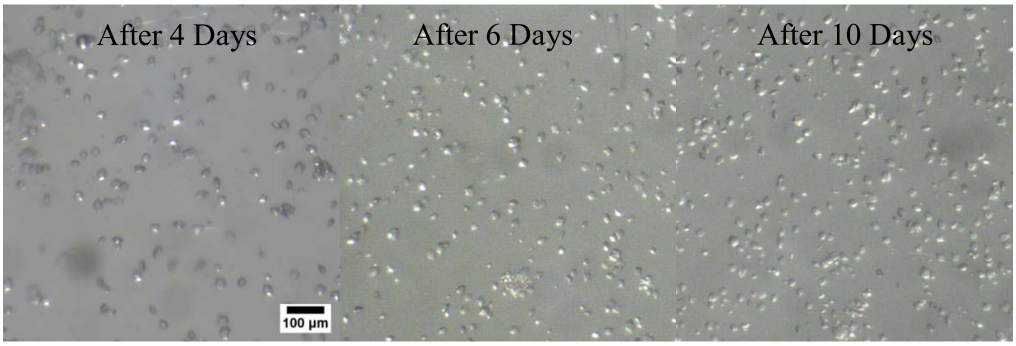

Figure S1 Series of microscopy images of lysozyme crystals grown in-situ. Crystals remained intact for over ten days.

S2.2. Crystal diffraction signal to noise

Figure S2 compares the signal to noise for the new chip for two randomly selected synchrotron X-ray shots, one with and one without diffraction. Gilbile et al. (2021) demonstrated that in-situ crystallization techniques could yield lower background when the crystals grow to span the spacer layer thickness. In this way, most of the crystallization buffer solution is excluded from the beam path. In other words, the crystals displace the crystallization buffer during growth, maximizing signal-to-noise. However, this requires in-situ grown crystal sizes to ideally match the spacer layer thickness, which might not be possible for some systems. When this is impossible, or with slurry-loaded crystals, background scattering from the remaining buffer remains the most significant contribution to the background.

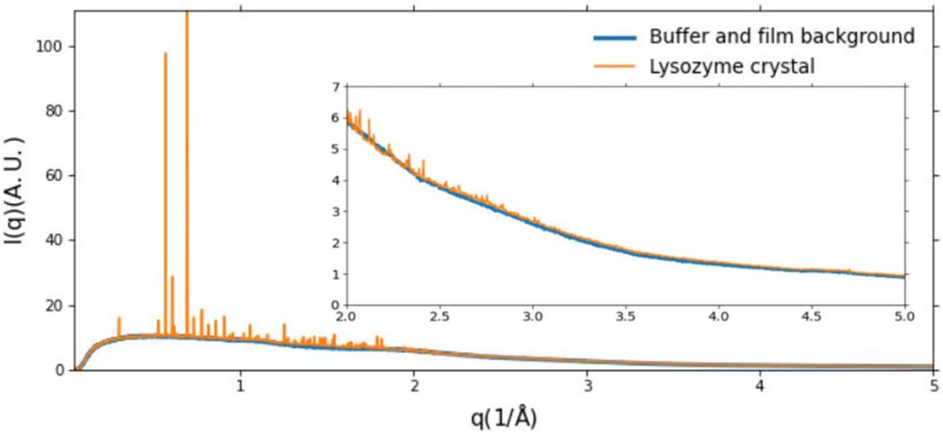

Figure S2 An exemplar radial scattering plot shows a lysozyme crystal's scatting intensity compared to background scattering from crystallization buffer and film. This plot demonstrates a chip with a 2.7 µm thick COC enclosing films with a flow layer thickness of 48 µm with scattering contributions from air subtracted. Distinguishable scattering peaks cease around q = 3 Å-1 in this example.

Table S1 shows the completeness of the XFEL lysozyme data. The high completeness in the low-resolution shells indicates that the data is free from high interference, overexposure, or map distortion.

Table S1 Completeness of the data over the entire resolution range.

Resolution	Reflections	Completeness
25.8393 - 9.4658	100/100	100.0%
9.4317 - 7.4129	100/99	100.0%
7.4031 - 5.8123	199/199	100.0%
5.8045 - 4.5632	395/395	100.0%
4.5594 - 3.5800	769/795	96.7%
3.5792 - 2.8103	1585/1585	100.0%
2.8094 - 2.2057	3237/3237	100.0%
2.2054 - 1.7312	6540/6544	99.9%
1.7311 - 1.3816	9469/12155	77.9%

Figure S3 shows in mean intensity curve calculated over each resolution bin. Deviation of the mean intensity from a typical protein sample indicates some background interferences likely from COC film at lower resolutions, but the high-resolution intensity was nearly unaffected. Interference at higher resolutions may also come from the sample buffer.

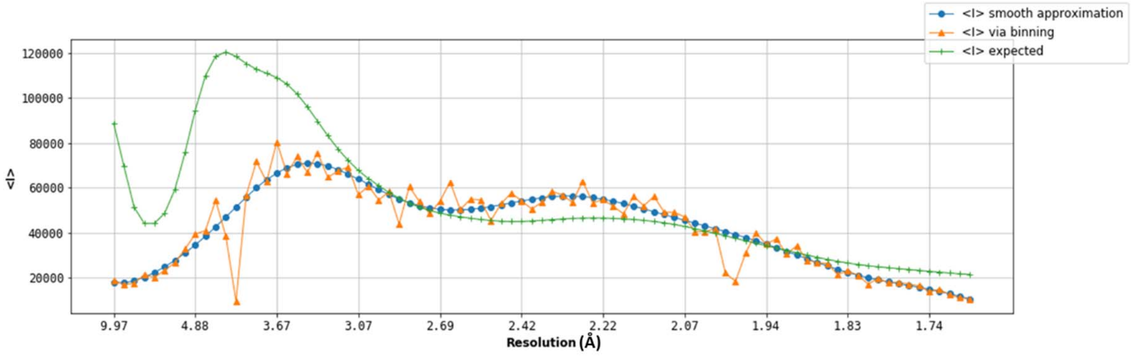

Figure S3 Mean intensity over the resolution range from Xtriage. The expected curve is an exemplar curve for a typical protein sample. The binning curve indicates the mean intensity over a step of the resolution bin.

Figure S4 shows how water permeability can be tuned by varying film thicknesses. Thicker films have lower water permeability and can meantime sample hydration for a longer time.

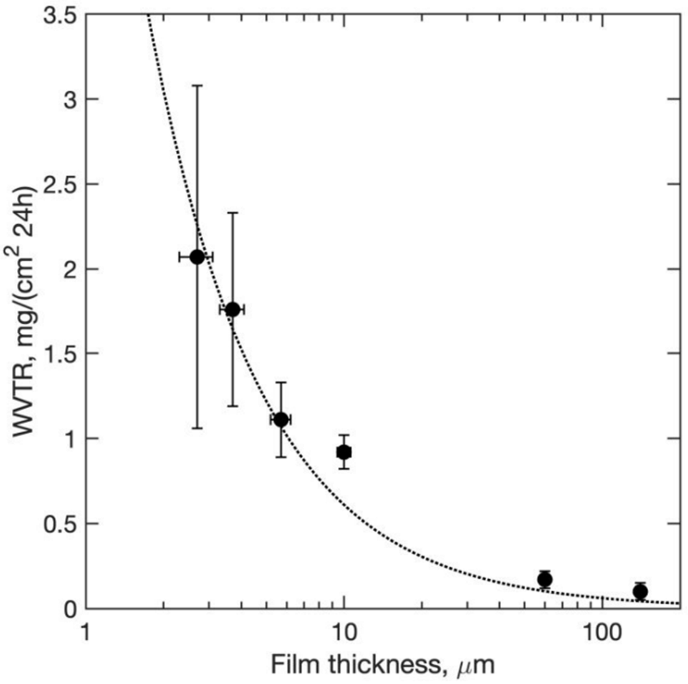

Figure S4 Steady-state water vapor transmission rates (WVTR) through COC films were measured using a modified wet-cup method at 23°C against a ΔRH gradient of approximately 80 percent. Adapted from D. Gilbile *et al*. 2021.
